# Incidence and outcomes of encapsulating peritoneal sclerosis (EPS) and factors associated with severe EPS

**DOI:** 10.1371/journal.pone.0190079

**Published:** 2018-01-02

**Authors:** Chin-Chung Tseng, Jin-Bor Chen, I-Kuan Wang, Shang-Chih Liao, Ben-Chung Cheng, An-bang Wu, Yu-Tzu Chang, Shih-Yuan Hung, Chiu-Ching Huang

**Affiliations:** 1 Division of Nephrology, Department of Internal Medicine, National Cheng Kung University Hospital, Tainan, Taiwan; 2 Department of Medicine, College of Medicine, National Cheng Kung University, Tainan, Taiwan; 3 Division of Nephrology, Department of Internal Medicine, Kaohsiung Chang Gung Memorial Hospital and College of Medicine, Chang Gung University, Kaohsiung, Taiwan; 4 Kidney Institute and Division of Nephrology, China Medical University Hospital and College of Medicine, China Medical University, Taichung, Taiwan; 5 Division of Nephrology, Department of Internal Medicine, E-DA Hospital, and School of Medicine for International Students, I-Shou University, Kaohsiung, Taiwan; University of Utah School of Medicine, UNITED STATES

## Abstract

**Background:**

Encapsulating peritoneal sclerosis (EPS) is a rare but serious complication of long-term peritoneal dialysis (PD). However, previous studies reported large variations in its mortality rates that may associate with a different degree of EPS severity. This study reports the incidence and outcomes of EPS and identifies the risk factors associated with severe EPS.

**Methods:**

We retrospectively analyzed clinical data of EPS patients from 3 medical centers in Taiwan from January 1982 to September 2015, and classified patients as having mild/moderate or severe EPS. Patients with intractable intestinal obstruction/gut-related sepsis that needed surgical intervention or resulted in mortality were in severe EPS group. Follow-up for outcome was through December 31, 2015. Clinical characteristics, peritoneal dialysis (PD)-related parameters, biochemical and imaging results were analyzed and compared between groups.

**Results:**

Fifty-eight of 3202 patients undergoing PD during the study period had EPS (prevalence 1.8%). The incidence of EPS increased for patients on PD for >6–8 years (≤6 yrs. vs. >6–8 yrs., 0.0% vs. 1.8%, *p* = 0.001). Relative to those on PD for >6–8 years, the risk of EPS significantly increased with PD duration longer than 10 years (>10–12 years *vs*. >6–8 years: OR: 5.5, 95% CI: 1.7–17.1, *p* < 0.01). Twenty-three patients fulfilled the criteria for severe EPS. The overall mortality rate of EPS was 35% (20/58), and was 74% (17/23) in the severe EPS group. The average serum levels of C-reactive protein (CRP) and intact-parathyroid hormone (i-PTH), which were checked every 3~6 months within one year before diagnosis of EPS, were higher in severe EPS group than in mild/moderate group (*p* = 0.02, *p* = 0.08, respectively). Multivariate analysis revealed severe EPS was independently associated with bowel tethering (based on CT), presentation with bloody ascites, diagnosis of EPS after withdrawal from PD, and i-PTH ≥ 384 pg/mL. Receiver operating characteristic analysis indicated that presentation with 2 or more of the 5 risk factors (EPS diagnosis after PD withdrawal, bloody ascites, bowel tethering, CRP ≥ 29 mg/L, and i-PTH ≥ 384 pg/mL) had a good accuracy (AUC = 0.80, *p* = 0.001) for prediction of severe EPS.

**Conclusions:**

The incidence of EPS increases with PD duration. Severe EPS has high mortality rate and is associated with bowel tethering, presentation of bloody ascites, diagnosis after PD withdrawal, and higher serum levels of i-PTH before EPS diagnosis. Having 2 or more of the 5 risk factors can provide a good accuracy for prediction of severe EPS.

## Introduction

Gandhi and colleagues first reported the presence of encapsulating peritoneal sclerosis (EPS) in 1980 [1]. Since then, EPS has been recognized as a rare but serious complication of long-term peritoneal dialysis (PD). EPS is characterized by microangiopathy, inflammation, and extreme peritoneal sclerosis, and leads to intestinal encapsulation and obstruction [2].

Data from EPS registries or study groups indicate that the prevalence of EPS is 0.5–2.5% among PD patients, and that the incidence increases with the duration of PD therapy [3–5]. An Australian registry study reported that the incidence of EPS was 19.4% after 8 years of PD treatment [3]. Kawanishi et al. performed a prospective study of PD patients in Japan, and reported the incidence of EPS was 0.7% after 5 years, 2.1% after 8 years, 5.9% after 10 years, and 17.2% after 15 years [5].

The effect of medical therapy for EPS is still unclear, but there are several non-evidence-based medical and surgical treatment options. Although there are currently no specific, evidence-based medical treatment options [6], the possible treatment regimens include immunosuppression or antifibrotic therapy with steroids, cyclosporine, and tamoxifen. Peritonectomy and enterolysis (PEEL) is the surgery of choice [7, 8].

Although EPS is the most serious complication of PD, previous studies reported large variations in the mortality rates ranging from 19% to 67% [3–5, 9–14], possibly because they studied patients with different severities. However, the factors associated with severe EPS are not yet well established. In addition, variations in the use of PD, withdrawal rate, and treatment duration among different countries could influence the reported incidences of EPS. To date, little is known about the incidence and outcome of EPS in Taiwan. In this study, we retrospectively analyzed the demographics, incidence, outcomes and clinical data of EPS patients from 3 tertiary medical centers in Taiwan between January 1982 and September 2015, and compared the characteristics of those who had severe EPS with those who had mild/moderate EPS to identify risk factors and predictors for severe EPS.

## Materials and methods

The study protocol was approved by the medical ethics committee of all 3 medical centers in Taiwan (National Chen Kung University Hospital, B-ER-104-069; China Medical University Hospital, CMUH103-REC2-070; and Kaohsiung Chang Gung Memorial Hospital, 100-2661B). All research ethics boards waived the requirement for individual informed consent for this retrospective study.

### Settings and patients

This multicenter retrospective-cohort study examined the records of all EPS patients diagnosed at 3 tertiary medical centers in Taiwan from January 1982 to September 2015. Follow-up continued until 31 December 2015. The PD unit of KCGMH has the largest PD patient number in Taiwan, and CMUH is the largest one in Central Taiwan, while NCKUH is the National Medical Center of Southern Taiwan. The PD patient numbers are 457, 393 and 161 in KCGMH, CMUH, and NCKUH in June 2015, respectively, accounting about 15.7% of total PD patients in Taiwan. (http://www.nhi.gov.tw)

### Diagnosis of EPS

The diagnostic criteria for EPS were from the *ad hoc* committee of the International Society for Peritoneal Dialysis (ISPD) [15], and the ISPD Working Party [16], and were mainly based on clinical symptoms and radiological findings [17]. EPS was suspected based on the following clinical symptoms: nausea, vomiting, fullness, absence of bowel sounds, abdominal pain, abdominal or pelvic mass, and other clinical aspects. The typical CT findings of EPS were peritoneal calcification, peritoneal thickening, bowel wall thickening, bowel tethering, lobulated ascites, and bowel dilatation.

### Definition of severe EPS and patient groups

We divided our EPS patients into 2 groups—severe disease or mild/moderate disease [9, 11]. Patients who had extensive symptoms and signs of intractable intestinal obstruction, gut ischemia, or gut-related sepsis, which needed surgical intervention or resulted in mortality were in the severe EPS group. All remaining EPS patients were in the mild/moderate EPS group.

### Factors and data collection

All clinical records were reviewed in detail, and individual data sheets were compiled. At EPS diagnosis, demographic data, cause of primary renal disease, and co-morbidities were recorded. The details of PD included PD duration, PD dose (L/day), icodextrin solution use, therapy modality at EPS diagnosis, last available residual renal Kt/V and peritoneal transport category and peritonitis rate (episode/patient-year) before EPS diagnosis.

The formula for calculating the incidence rate of EPS for the indicated duration was **A**/**B**. **A** is the total EPS patient number, and **B** is the total PD patient number in the relevant interval of PD duration (e.g. PD duration ≤ 6 years, or others).

Biochemical profiles including serum levels of albumin, C-reactive protein (CRP), intact parathyroid hormone (i-PTH), corrected calcium, phosphate, and blood hemoglobin (Hb) were collected. The value of each biochemical parameter was the mean of all the data checked every 3 to 6 months within 1 year before the diagnosis of EPS. Plain abdominal film and computed tomography at EPS diagnosis were reviewed to assess correlations with EPS severity. The EPS characteristics included status at EPS onset, cause of PD withdrawal before EPS diagnosis, status at last follow-up, outcome, and cause(s) of mortality.

### Statistical analysis

Results are expressed as numbers and percentages for categorical variables, as means ± standard deviations (SDs) for normally distributed continuous variables, and as medians and ranges or interquartile ranges for non-normally distributed continuous data. The differences between severe and mild/moderate EPS patients were analyzed using the chi-square test or Fisher’s exact test for categorical data, as appropriate, an unpaired *t*-test for continuous normally distributed data, and a Mann-Whitney test for non-normally distributed data. Each variable with a *p* value less than 0.1 in univariate analyses was included in the multivariate binary logistic regression analysis. Receiver operating characteristic (ROC) analysis, with an estimate of area under the ROC curve (AUC), was used to identify significant predictors of severe EPS. A *p*-value below 0.05 was considered statistically significant. Statistical analysis was performed using SPSS for Windows, version 17.0.

## Results

### Demographics, incidence and clinical characteristics of EPS

**Fig 1:**
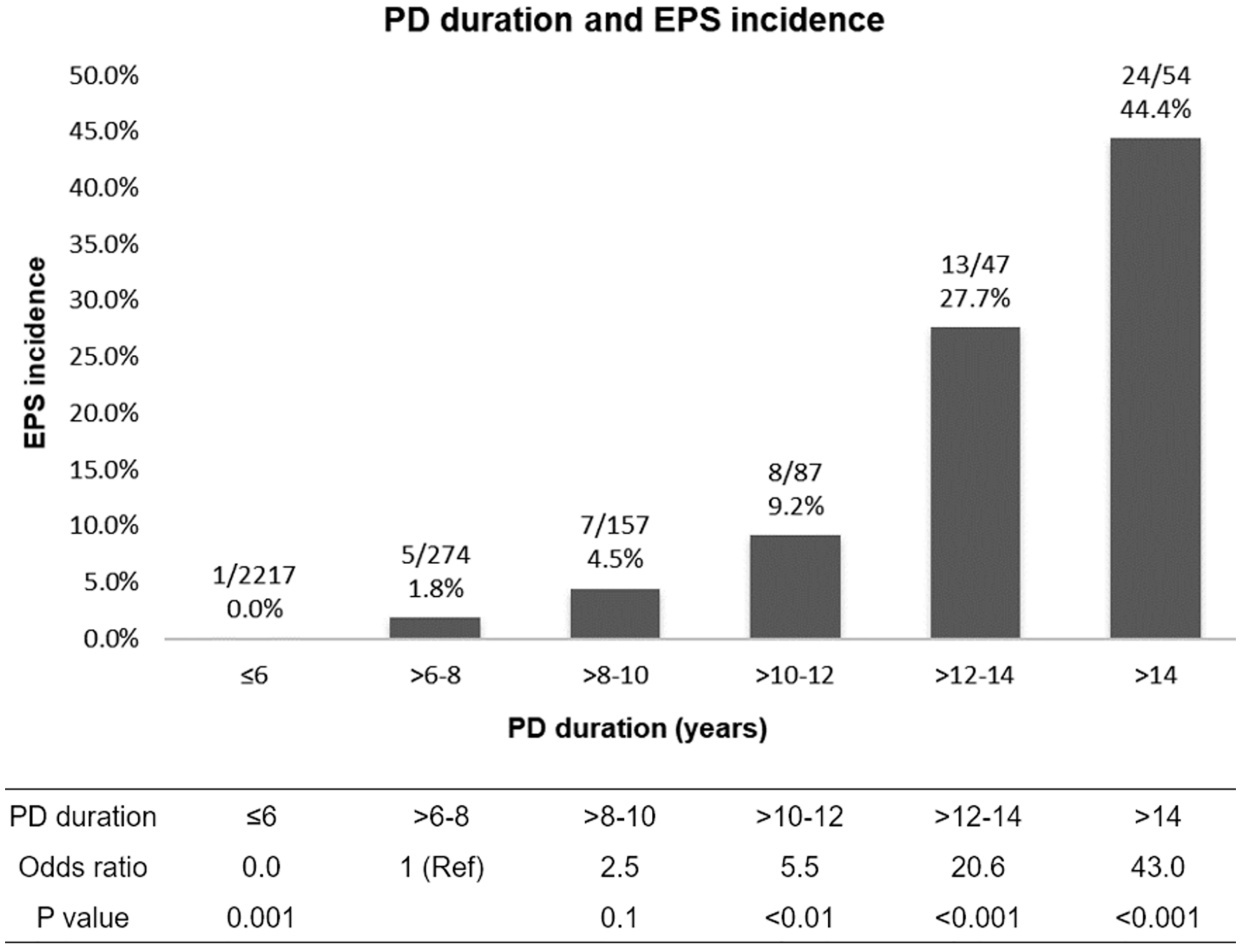
Effect of PD duration on the incidence rate of EPS. The number of EPS patients and total number of patients who received PD for the indicated duration is on the top of each bar. Incidence rates were calculated based on 2836 PD patients for whom PD duration was known (2778 non-EPS patients and 58 EPS patients). The incidence of EPS for patients on PD for >6–8 years was similar to that of patients on PD for >8–10 years (1.8% Vs. 4.5%, odds ratio [OR]: 2.5, *p* = 0.1). Relative to those on PD for >6–8 years, the risk of EPS increased with PD duration (OR: 5.5, 95% CI: 1.7–17.1, for >10–12 years; OR: 20.6, 95% CI: 6.9–61.3, for >12–14 years; OR: 43.0, 95% CI: 15.3–121.1, for more than 14 years).

There were 3202 patients undergoing PD at the 3 medical centers in Taiwan from January 1982 to December 2015. During this study period, 58 patients developed EPS, corresponding to an overall prevalence of 1.8%. The mean annual incidence rate of EPS was 2.6 per 1000 patient-years of PD, with a range 0 to 15. There were 20 (35%) men and 38 (66%) women, with a mean age of 50.4 ± 11.6 years (range: 30 to 80) (Table 1). Fig 1 shows the relationship of the incidence rate of EPS with PD duration. We calculated these incidence rates for all 2836 PD patients whose PD durations were recorded (2778 non-EPS patients and 58 EPS patients). Only one EPS case was on PD for less than 6 years. The percentage of EPS increased for patients on PD for 6–8 years (≤6 yrs. vs. >6–8 yrs., 0.0% vs. 1.8%, *p* = 0.001). The percentage of EPS was 4.5% for patients on PD for >8–10 years which was similar to that of patients on PD for >6–8 yrs. (odds ratio [OR]: 2.5, *p* = 0.1). The percentage of EPS cases was 9.2% for those on PD for >10–12 years, 27.7% for those on PD for >12–14 years, and 44.4% for those on PD more than 14 years. Relative to those on PD for >6–8 years, the risk of EPS significantly increased with PD duration longer than 10 years. (Fig 1).

**Table 1:** Clinical characteristics of EPS patients.

Clinical characteristic	Total (N = 58)^a^	Severe EPS (n = 23)	Mild/moderate EPS (n = 34)	P value^b^
**Age at start of PD, years**	38.5 ± 13.2	37.4 ± 12.6	39.1 ± 13.8	0.6
**Age at EPS diagnosis, years**	50.4 ± 11.6	50.3 ± 13.0	52.1 ± 10.8	0.6
**Female sex**	38 (66)	15 (65)	23 (68)	0.9
**ESRD etiology**				0.1
DM nephropathy	9 (16)	3 (13)	6 (18)	
Hypertension	1 (2)	0 (0)	1 (3)	
CGN	33 (57)	13 (57)	19 (56)	
CTIN	4 (7)	4 (17)	0 (0)	
Polycystic kidney disease	1 (2)	1 (4)	0 (0)	
Lupus nephritis	1 (2)	0 (0)	1(3)	
Others	2 (3)	0 (0)	2 (6)	
Unknown	7 (12)	2 (9)	5 (15)	
**Comorbidity**				
Diabetes mellitus	11 (19)	4 (17)	7 (21)	1.0
Hypertension	28 (48)	14 (61)	14 (41)	0.2
Cardiovascular disease	6 (10)	2 (9)	4 (12)	1.0
Malignancy	6 (10)	4 (17)	2 (6)	0.2

Data are presented as means ± SDs or numbers (percentages).

Abbreviations here and below: PD, peritoneal dialysis; EPS, encapsulating peritoneal sclerosis; ESRD, end stage renal disease; DM, diabetes mellitus; CGN, chronic glomerulonephritis; CTIN, chronic tubulointerstitial nephritis.

^a^One patient with unknown cause of death was not included in either group.

^b^P for severe group *vs*. mild/moderate group.

### Groups of EPS patients

Twenty-three of the 58 patients fulfilled the criteria for severe EPS; eight of these patients received surgery and the other 15 patients did not receive surgical interventions because of the high risk of surgical complications.

We classified the other 34 patients as having mild/moderate EPS. These patients presented with the signs and symptoms of EPS (such as reduced ultrafiltration, high peritoneal transport state, hypoalbuminemia, ascites, poor appetite and intermittent gastrointestinal obstruction), but could be controlled by aggressive medical management and survived without surgical intervention. The diagnosis of EPS in these patients was confirmed by clinical manifestations and computed tomographic imaging to assess the presence of peritoneal thickening, peritoneal calcification, loculated ascites, and encapsulated bowel.

One additional patient, with unknown cause of death, was not included in either group. The severe and mild/moderate groups had similar follow-up durations (12.6 months (IQR: 6.5, 27.5) *vs*. 16.0 months (IQR: 9.5, 31.0), *p* = 0.6).

### PD-related clinical parameters

The mean duration of PD in all 58 EPS patients was 13.2 years, and the range was 4.8 to 21.7 years (Table 2). Analysis of the peritoneal transport characteristics indicated that most patients were high or high-average transporters (n = 51, 93%) (Table 2). The median peritonitis rate of all patients was 0.08 episode/patient-year (Table 2), and the average was 0.14 episode/patient-year. These are lower than the overall peritonitis rate of the 3 centers over the matched period (0.22 episode/patient-year). The 2 groups had no significant difference in PD duration, peritoneal transport characteristics, PD dose, and peritonitis rate.

**Table 2:** Peritoneal dialysis-related characteristics in EPS patients.

PD characteristic	Total (N = 58)^b^	Severe group (n = 23)	Mild/moderate group (n = 34)	P value^c^
**PD duration, years**	13.2 ± 3.8	13.0 ± 3.3	13.4 ± 4.1	0.7
**PD modality, CAPD/APD**^a^	52/6	21/2	30 /4	1.0
**Peritoneal transport category**				
High average/High	51 (93)	19 (95)	31 (91)	1.0
**PD dose, L/day**	9.2 ± 2.3	9.1 ± 2.2	9.0 ± 2.1	0.8
**Icodextrin use**	50 (89)	19 (86)	30 (91)	0.7
**Duration of icodextrin use, months**	78.6 (22.0, 103.4)	67.0 (33.0, 94.0)	88.3 (17.5, 104.1)	0.9
**Renal Weekly Kt/V**	0 (0, 0)	0 (0, 0)	0 (0, 0)	0.9
**Peritonitis rate before EPS, episode/patient-year**	0.08 (0, 0.21)	0.07 (0, 0.20)	0.10 (0.05, 0.37)	0.1

Data are presented as means ± SDs (normally distributed data), medians (interquartile ranges) (non-normally distributed data), or numbers (percentages).

^a^PD modality at EPS diagnosis: automatic peritoneal dialysis (APD) or continuous ambulatory peritoneal dialysis (CAPD).

^b^One patient with unknown cause of death was not included in either group.

^c^P for severe group *vs*. mild/moderate group.

### Biochemical and imaging results

The 2 groups had no significant difference in biochemical parameters, except for serum levels of C-reactive protein (CRP), which were higher in the severe EPS group (*p* = 0.02) (Table 3). The serum level of intact-parathyroid hormone (i-PTH) was higher in the severe EPS group, but with a borderline significance (*p* = 0.08). Computed tomography (CT) was available for 57 of the 58 patients. The most common CT finding was peritoneal calcification (49 of 57, 86%). Bowel tethering (based on CT) was significantly more common in patients with severe EPS than mild/moderate EPS (61% *vs*. 24%, *p* < 0.01).

**Table 3:** Biochemical profiles and imaging findings.

Characteristic	Total (N = 58)^d^	Severe group (n = 23)	Mild/moderate group (n = 34)	P value^e^
**Biochemical parameter**^a^				
Albumin, g/dL	3.7 ± 0.4	3.7 ± 0.4	3.7 ± 0.4	0.8
CRP, mg/L	17 (5, 44)	40 (12, 52)	13 (4, 32)	0.02
i-PTH, pg/mL	276 (130, 533)	408 (215, 646)	209 (81, 495)	0.08
Corrected Calcium, mg/dL	9.6 ± 1.0	9.7 ± 0.8	9.5 ± 1.0	0.4
Phosphate, mg/dL	4.9 ± 1.0	5.1 ± 0.9	4.8 ± 1.0	0.4
Hb, g/dL	10.0 ± 1.4	9.6 ± 1.6	10.2 ± 1.2	0.1
**Plain abdominal film**^b^				
Curvilinear peritoneal calcification	34 (62)	11 (52)	23 (68)	0.3
**Computed tomography**^c^				
Peritoneal thickening	34 (60)	14 (61)	20 (59)	0.9
Peritoneal Calcification	49 (86)	21 (91)	28 (82)	0.5
Ascites	37 (65)	16 (70)	21 (62)	0.6
Bowel dilatation	17 (30)	7 (30)	10 (30)	0.9
Bowel tethering	22 (39)	14 (61)	8 (24)	<0.01

Data are presented as means ± SDs (normally distributed data), medians (interquartile ranges) (non-normally distributed data), or numbers (percentages).

Abbreviations here and below: CRP, C-reactive protein; Hb, hemoglobin; i-PTH, intact parathyroid hormone.

^a^Values of biochemical parameters were the mean of the data checked every 3 to 6 months within one year before EPS diagnosis.

^b^Plain abdominal film images were available for 55 patients.

^c^Computed tomography images were available for 57 patients.

^d^One patient with unknown cause of death was not included in either group.

^e^P for severe group *vs*. mild/moderate group.

### EPS characteristics

Bloody ascites (or bloody dialysate effluent when PD was ongoing) was more common in patients with severe EPS (61% *vs*. 30%, *p* = 0.02, Table 4). Twenty-six of all 58 patients (45%) developed EPS after withdrawal from PD, and withdrawal was a more common antecedent of EPS in patients with severe EPS (65% *vs*. 32%, *p* = 0.02, Table 4). The causes of PD withdrawal were similar in the 2 groups (Table 4). The median duration of EPS onset from PD withdrawal was 3.7 months for the severe group, and 2.5 months for the mild/moderate group, with no significant difference (*p* = 0.4).

**Table 4:** Characteristics of EPS and patient outcomes.

Characteristic	Total (N = 58)^a^	Severe group (n = 23)	Mild/moderate group (n = 34)	P value^b^
**Presented with bloody ascites**	25 (44)	14 (61)	10 (30)	0.02
**EPS onset after PD withdrawal**	26 (45)	15 (65)	11 (32)	0.02
**Causes of PD withdrawal**				
Refractory peritonitis	16 (62)	7 (47)	9 (82)	0.1
Non-peritonitis	10 (39)	8 (53)	2 (18)	0.1
UF failure or inadequate dialysis	5 (19)	4 (27)	1 (9)	
Refractory tunnel infection	1 (4)	1 (7)	0 (0)	
Abdominal surgery	1 (4)	1 (7)	0 (0)	
Hernia	1 (4)	1 (7)	0 (0)	
Other	2 (8)	1 (7)	1 (9)	
**Duration from PD withdrawal to EPS onset, months**	3.7 (1.9, 6.2)	3.7 (1.9, 6.9)	2.5 (1.6, 6.4)	0.4
**Outcome**				
**Mortality**	20 (35)	17 (74)	2 (6)	<0.01
**Causes of mortality**				
Bowel perforation	9 (50)	9 (53)	0 (0)	
Peritonitis	2 (11)	2 (12)	0 (0)	
Malnutrition/cachexia	2 (11)	2 (12)	0 (0)	
Vascular access infection	1 (5)	1 (6)	0 (0)	
Pneumonia/respiratory failure	2 (11)	2 (6)	0 (0)	
Sepsis	1 (5)	1 (6)	0 (0)	
Acute myocardial infarction	1 (5)	0 (0)	1 (50)	
Sepsis after heart surgery	1 (5)	0 (0)	1 (50)	
Unknown	1 (5)	0 (0)	0 (0)	
**Duration from EPS diagnosis to mortality, months**	14.9 (9.1, 31.4)	14.4 (5.9, 31.3)	27.5 (17, NA)	0.4

Data are presented as means ± SDs (normally distributed data), medians (interquartile ranges) (non-normally distributed data), or numbers (percentages).

UF, ultrafiltration failure.

^a^One patient with unknown cause of death was not included in either group.

^b^P for severe *vs*. mild/moderate group.

### Outcomes

EPS patients were initially controlled by medical management, including nutrition support, ascites drainage, and medications (steroids, tamoxifen, or both if there were no contraindications). Surgical intervention was not needed for any of the patients with mild/moderate EPS, but 2 of these patients died of other causes (acute myocardial infarction and sepsis after heart surgery).

For patients with severe EPS, the symptoms progressed or persisted, and 8 of these patients received surgical enterolysis. The median duration from EPS diagnosis to surgery was 5.5 months (interquartile range [IQR]: 1.1, 19.7). Two of these 8 patients died (8.8 and 3.2 months after surgery), because of bowel leakage with peritonitis and sepsis; the other 6 patients (75%) were alive at the end of study (December 31, 2015). The median follow-up duration from surgery to mortality or study end was 8.8 months (IQR: 3.1, 16.3). The other 15 patients with severe EPS who did not receive operations eventually died of EPS-related complications.

Totally, 20 patients (17 in the severe group, 2 in the mild/moderate group, 1 with unknown cause of death who was not included in either group) died, corresponding to an overall mortality rate of 35% (Table 4). The mortality rate (74%) was much greater in patients with severe EPS (*p* < 0.01). The causes of death in patients with severe EPS were bowel perforation or leakage (n = 9, 53%), peritonitis (n = 2, 12%), malnutrition (n = 2, 12%), and infection/sepsis (n = 4, 24%) (Table 4). Among patients who died, the overall median survival duration was 14.9 months (IQR: 9.1, 31.4), and the median survival time of those with severe EPS was 14.4 months (IQR: 5.9, 31.3).

### Multivariate analyses of factors associated with severe EPS

We derived ORs from 2 separate conditional logistic models (Table 5). In model 1, we included CRP and i-PTH as continuous variables; in model 2, we included 2 categories each of CRP and i-PTH, using the mean levels of all 57 EPS patients as the cut-offs. The results of model 1 indicate that EPS onset after PD withdrawal, presence of bloody ascites and higher serum level of i-PTH were significantly associated with severe EPS. The results of model 2 indicate that EPS onset after PD withdrawal, presence of bloody ascites, bowel tethering, bloody ascites and serum level of i-PTH above 384 pg/mL were significantly associated with severe EPS. Serum level of CRP is higher in severe EPS group, but it was not an independent risk factor.

**Table 5:** Multivariate analysis of factors associated with severe EPS.

Variable	OR^a^ (95% CI)	P value	OR^b^ (95% CI)	P value
**EPS onset after PD withdrawal**	8.5 (1.1, 65.3)	0.04	7.6 (1.2, 46.7)	0.03
**Bloody ascites**	8.0 (1.2, 54.5)	0.03	6.6 (1.1, 39.5)	0.04
**Bowel tethering**	6.7 (0.8, 55.2)	0.08	7.6 (1.1, 51.0)	0.04
**CRP level (mg/L)**^a^	1.0 (1.0, 1.1)	0.13		
**i-PTH level (pg/mL)**^a^	1.0 (1.0, 1.01)	0.02		
**CRP < 29**^b^			1	Ref.
**CRP ≥ 29**^b^			1.8 (0.3, 9.8)	0.5
**i-PTH < 384**^b^			1	Ref.
**i-PTH ≥ 384**^b^			7.4 (1.0, 54.0)	0.049

Odds ratios were derived from 2 conditional logistic models:

^a^Model 1: CRP and i-PTH values were included as continuous variables;

^b^ Model 2: CRP and i-PTH values were divided into 2 groups, in which each cut-off point was the mean level among all 57 EPS patients who were included for analysis.

Based on AUC values, each of these individual parameters could be considered “poor” or “fair” predictors of EPS severity (*i*.*e*. AUC 0.6 to 0.7) (S1 Table). Thus, we evaluated the use of multiple parameters. The results show that the presence of 2 or more of the 5 risk factors (EPS onset after PD withdrawal, presentation of bloody ascites, bowel tethering, CRP ≥ 29 mg/L, and i-PTH ≥ 384 pg/mL) has better result with a good discriminability (AUC = 0.80, *p* = 0.001, sensitivity = 94%, specificity = 67%, S1 Table) for prediction of severe EPS.

## Discussion

This study is the largest multicenter series in Taiwan to provide detailed information about patients with EPS. We present the incidence, clinical findings, PD details and outcomes of 58 EPS patients from 3 medical centers. This study is important because it provides an additional experience on a very rare disease. In addition, we divided our patients into 2 groups—severe EPS and mild/moderate EPS—to identify the risk associated with severe EPS.

In our series, there were 23 severe EPS patients, 8 patients received surgical interventions, 2 of whom died, and 15 non-surgical patients also died, corresponding to a mortality rate of 74% (17/23). Similarly, Summer et al. also revealed a high mortality rate in their severe EPS patients [9]. Thirteen of their 16 severe cases received operations; 5 patients who received surgery and 3 who did not die, corresponding to a mortality rate of 50% for patients with severe EPS. Both the series demonstrated that severe EPS had a poor outcome with high mortality rate.

The overall incidence of EPS in this multicenter survey was 1.8% which is like the results reported by other series [3–5, 9–14]. However, we demonstrate that EPS is very rare (only 1 case among 2217 patients) for patients on PD for less than 6 years. The risk of developing EPS increased for patients on PD for 6–10 years, but was not different between on PD for 6–8 and 8–10 years; a significant increase in the risk of developing EPS was found for patients on PD for more than 10 years. Thus, EPS should be considered a possibility for patients on PD for 6 or more years, and more careful evaluation is needed for those on PD for 10 or more years. Our finding of a very high incidence of EPS in patients on PD for more than 14 years may be an overestimation, due to the relatively small number of patients who received PD for such a long time.

Peritonitis episodes have long been considered risk factors for EPS development [3, 18, 19]. However, our study revealed that EPS patients had a lower peritonitis rate than all other non-EPS patients during the matched period (0.14 vs. 0.22 episode/patient-year). Similarly, several studies also could not confirm an association between the incidence of peritonitis and EPS [10, 20, 21]. Forty-three (74%, 43/58) of our EPS patients had a PD duration longer than 10 years. These patients can maintain on PD for a longer duration because they had a lower incidence of peritonitis and peritonitis-related withdrawal from PD. A prospective study in Japan also showed that bacterial peritonitis was less directly associated with EPS development in patients with PD duration of more than 10 years, because there were originally few episodes of peritonitis in these patients [5].

Our results revealed that a higher serum level of CRP in the severe EPS group, although CRP was not an independent factor, and that onset of EPS after discontinuation of PD was associated with severe EPS. The presence of inflammation is essential for the development of EPS, because the initial components of the encapsulating peritoneum are inflammatory products, such as fibrin [22]. Thus, the higher serum levels of CRP may indicate greater inflammation in the damaged peritoneum, which leads to increased loss of fibrin into the peritoneal cavity, and induces more severe intestinal adhesion and encapsulation. Fibrin deposited during PD is washed away by the daily dialysate drainage. After cessation of PD, the peritoneal cavity retains fibrin, and this accelerates encapsulation [7]. Thus, strict monitoring is necessary when a patient is withdrawn from long-term PD. The reasons for cessation of PD in severe EPS patients included refractory peritonitis, ultrafiltration failure, or inadequate dialysis, which may be the early symptoms/signs of EPS itself. Therefore, we cannot exclude the possibility of a delayed diagnosis in some of these patients. Nonetheless, EPS onset after PD withdrawal may be an important risk factor for severe EPS, independent of the reasons for PD withdrawal.

Our study demonstrated that presentation with bloody ascites was associated with severe EPS. Overall, 25 (44%) of our EPS patients presented with bloody ascites. Previous studies also reported bloody ascites in 7–50% of EPS patients [3, 23], and that this condition usually appears in early-stage EPS [22–25]. In fact, bloody ascites may occur from early- to late-stage EPS [24]. The appearance of bloody ascites may be a consequence of the inflammatory nature of this disease, in which peritoneal neoangiogenesis is prominent [15], and there is increased probability of bleeding. Peritoneal bleeding may further exacerbate the inflammatory response, and facilitate the process of adhesion [15, 26].

We also found that the serum level of i-PTH was significantly associated with severe EPS in multivariate analysis. A higher serum level of i-PTH is a consequence of more severe renal hyperparathyroidism, and may increase the intensity of peritoneal calcification in long-term PD patients who have been exposed to hypertonic dialysate, repeated peritonitis, or excessive vitamin D therapy [27–29]. Previous surgical findings indicated that calcification is common around blood capillaries in the damaged peritoneal tissue, and sometimes extends from the serous layer into the muscular layer [22]. Furthermore, in the calcified region, severe adhesion to capsules frequently develops into a bowel obstructing lesion, and makes identification of the ablation layer difficult, thus increasing the risk of bowel perforation during surgery [22].

Tarzi et al. [17, 30] and Vlijm et al. [30] assessed the validity of an abdominal computed tomography (CT) scoring system for the diagnosis of EPS. Their results showed a significant difference of total CT scores between those with EPS and controls who were on PD or hemodialysis. Bowel tethering and peritoneal calcification were the most specific parameters [17]. However, CT score did not correlate with clinical outcome (death, surgery, or prolonged parental nutrition) [17]. In our study, bowel tethering was more common in patients with severe EPS. Recently, Kitterer et al. [31] demonstrated that CRP level and an abdominal CT scan are useful tools to predict the macroscopic appearance and to identify EPS patients with higher risk of complications prior to surgery.

Our ROC analysis indicated that any single parameter only provided “poor” or “fair” discriminability for prediction of severe EPS (*i*.*e*. AUC 0.6 to 0.7). However, we found that patients with 2 or more of the 5 risk factors (EPS onset after PD withdrawal, presentation of bloody ascites, bowel tethering on CT image, CRP ≥ 29 mg/L, and i-PTH ≥ 384 pg/mL) can provide a better result with good discriminability (AUC = 0.80, *p* = 0.001, sensitivity = 94%, specificity = 67%) for prediction of severe EPS.

The major limitations of this study are that it was retrospective and data was unavailable for some patients. Another limitation of this study is that data were from a 33-year period, so there could have been changes in diagnosis and treatment of EPS over this time.

In summary, the risk of EPS was greater in patients who were on PD for more than 6–8 years, and the risk increased further in patients who were on PD duration for more than 10 years. Our finding of higher serum levels of CRP in patients with severe EPS corroborates previous reports. The novel findings of this study are that severe EPS is significantly and independently associated with a diagnosis after withdrawal from PD, presentation of bloody ascites, bowel tethering on CT (in model 2) and higher serum levels of i-PTH. Patient with 2 or more of these 5 risk factors provided a better discriminability and good accuracy for prediction of severe EPS. Each of these factors is an indication of the underlying intensity of EPS. The results of our study highlight the important role of these signs and symptoms for the early assessment of EPS severity, and provide important information for management of EPS.

## Supporting information

S1 Table ROC analysis of the accuracy of predicting severe EPS with different risk factors. (PDF)

S1 Dataset Data for Fig 1. (XLSX)

S2 Dataset Dataset for Tables 1 to 5 and S1 Table. (XLS)
