# Supplementary material for: Incidence and outcomes of encapsulating peritoneal sclerosis (EPS) and factors associated with severe EPS
Source: PLoS One. 2018 Jan 2;13(1):e0190079. doi: 10.1371/journal.pone.0190079 (PMC5749741; doi:10.1371/journal.pone.0190079)
Supplement: S1 Table — (PDF) [file pone.0190079.s001.pdf]

S1 Table. ROC analysis of the accuracy of predicting severe EPS with different risk factors

Risk factor	AUC (95% CI)	P value	Sensitivity	Specificity
EPS onset after PD withdrawal	0.66 (0.52, 0.81)	0.04	0.65	0.68
Bloody ascites	0.65 (0.50, 0.80)	0.05	0.61	0.7
Bowel tethering	0.69 (0.54, 0.83)	0.02	0.61	0.76
CRP ≥ 29 mg/L	0.67 (0.51, 0.83)	0.05	0.59	0.75
i-PTH ≥ 384 pg/mL	0.56 (0.41, 0.72)	0.4	0.5	0.63
Number of risk factors[a]				
any one risk factor	0.58 (0.42, 0.75)	0.4	1.00	0.17
2 or more risk factors	0.80 (0.68, 0.93)	0.001	0.94	0.67

ROC, receiver operating characteristic; AUC, area under ROC curve.

[a]Risk factors: EPS onset after PD withdrawal, Bloody ascites, Bowel tethering, CRP ≥ 29 mg/L, i-PTH ≥ 384 pg/mL.
